# Elevated Mitochondrial Oxidative Stress Impairs Metabolic Adaptations to Exercise in Skeletal Muscle

**DOI:** 10.1371/journal.pone.0081879

**Published:** 2013-12-06

**Authors:** Justin D. Crane, Arkan Abadi, Bart P. Hettinga, Daniel I. Ogborn, Lauren G. MacNeil, Gregory R. Steinberg, Mark A. Tarnopolsky

**Affiliations:** 1 Department of Kinesiology, McMaster University, Hamilton, Ontario, Canada; 2 Department of Pediatrics, McMaster University, Hamilton, Ontario, Canada; 3 Department of Medical Sciences, McMaster University, Hamilton, Ontario, Canada; 4 Department of Medicine, McMaster University, Hamilton, Ontario, Canada; INSERM/UMR 1048, France

## Abstract

Mitochondrial oxidative stress is a complex phenomenon that is inherently tied to energy provision and is implicated in many metabolic disorders. Exercise training increases mitochondrial oxidative capacity in skeletal muscle yet it remains unclear if oxidative stress plays a role in regulating these adaptations. We demonstrate that the chronic elevation in mitochondrial oxidative stress present in *Sod2* ^*+/-*^ mice impairs the functional and biochemical mitochondrial adaptations to exercise. Following exercise training *Sod2* ^*+/-*^ mice fail to increase maximal work capacity, mitochondrial enzyme activity and mtDNA copy number, despite a normal augmentation of mitochondrial proteins. Additionally, exercised *Sod2* ^*+/-*^ mice cannot compensate for their higher amount of basal mitochondrial oxidative damage and exhibit poor electron transport chain complex assembly that accounts for their compromised adaptation. Overall, these results demonstrate that chronic skeletal muscle mitochondrial oxidative stress does not impact exercise induced mitochondrial biogenesis, but impairs the resulting mitochondrial protein function and can limit metabolic plasticity.

## Introduction

Mitochondria are a major source of reactive oxygen species (ROS) and reactive nitrogen species (RNS) and complexes I, II and III can release the radical superoxide during electron transfer [1-3]. The accumulation of oxidatively modified proteins, lipids and DNA resulting from spontaneous reaction with superoxide and other ROS/RNS is proposed to contribute to the mitochondrial dysfunction associated with a host of health problems including insulin resistance [4], neurodegeneration [5] and sarcopenia [6]. Post-mitotic tissues such as skeletal muscle and brain are particularly susceptible to mitochondrial oxidative damage because they are terminally differentiated, have a relatively slow cellular turnover and a high metabolic rate. However, many health interventions that intend to augment mitochondrial content and metabolism in differentiated tissues in order to counter disease progression must contend with an environment of chronic mitochondrial oxidative stress (i.e. aging, obesity). Therefore, insight regarding the consequences of mitochondrial oxidative damage is necessary in order to understand its broader role in altering mitochondrial function and adaptation. 

Habitual exercise is well known to improve health and enhance the mitochondrial content and oxidative capacity of skeletal muscle. Exercise training up-regulates antioxidant enzymes [7], presumably in response to increased ROS generation. Mitochondrial ROS, however, are not believed to contribute significantly to contraction-induced ROS production in skeletal muscle [8], and there is a lack of understanding with regard to exercise adaptation under conditions of mitochondrial oxidative stress. It is hypothesized that a greater proportion of mitochondrial superoxide is generated under basal (state 4) conditions than with high flux (state 3) mitochondrial respiration [9], but it remains unknown whether exercise training alters basal ROS generation in parallel to the increase in mitochondrial mass. In conditions where persistent oxidative stress accompanies disease progression, such as chronic obstructive pulmonary disease (COPD), exercise interventions appear to increase levels of oxidative damage in skeletal muscle [10] by increasing mitochondrial ROS [11]. Dietary antioxidant compounds have been utilized to elucidate the role of ROS in mediating skeletal muscle adaptations, with evidence that antioxidants can blunt the positive metabolic benefits of exercise within muscle [12,13]; however, there remains uncertainly regarding these effects [14]. Part of the controversy likely relates to variable uptake of these compounds in different tissues, non-specific cellular compartmentalization and complex dosage dependent pro-oxidant/anti-oxidant properties that are not clear *in vivo* (e.g. as with vitamin C, [15]). In relation to this ambiguity, the consumption of some antioxidant compounds may actually be harmful [16]. 

Given the importance of mitochondrial function to human health and the controversial role(s) of ROS in mediating adaptation and pathology, it is necessary to clarify how exercise, mitochondrial function and oxidative stress interact within skeletal muscle to regulate metabolic adaptation. We chose to use the *Sod2* ^*+/-*^ mouse as this strain does not exhibit any baseline impairment in physical function or lifespan [17] (limiting the influence of developmental defects) and has a similar reduction in muscle Sod2 activity as found in human disorders associated with oxidative stress (aging [18], obesity [19], type 2 diabetes [20]). Furthermore, mitochondrial superoxide specifically appears to be critical for cellular function as this enzyme is essential for life beyond perinatal development in mice [21,22], in contrast to the antioxidant enzymes Sod1, Gpx1 or Gpx4 [23-25]. 

## Materials and Methods

### Animal Handling and Care

*Sod2* ^*+/-*^ male and female mice were acquired from Jackson Laboratories and bred to produce *Sod2* ^*+/-*^ and *Sod2* ^*+/+*^ littermates. Mice were housed in micro-isolator cages and maintained under controlled environmental conditions (12/12 h light/dark cycle, 23°C). Mice received chow food (Diet 8640, Harlan Teklab, Madison, WI) and water *ad libitum*. Food intake and body weight were recorded biweekly during the study. At 6 months of age, mice were randomly allocated into sedentary or forced-endurance exercise training interventions for 16 weeks. At ~10 months of age all mice were sacrificed, after an overnight fast, by cervical dislocation. Sacrifice was performed one week following any *in vivo* testing procedures and the final exercise bout. All animal procedures were conducted under the approval of McMaster University’s Animal Research Ethics Board and the Canadian Council on Animal Care.

### Exercise Training Protocol

Mice in the exercise groups ran on level treadmills (Eco 3/6 treadmill; Columbus Instruments, Columbus, OH, USA) three times per week for 16 weeks. Initially the speed was set at 12 meters/minute (m/min) for 30 minutes, but gradually increased to 20 m/min for 60 minutes by the conclusion of the study. Mice were encouraged to run by the use of electrical shock bars at the ends of the treadmill lanes. All mice had similar adherence to the training regimen and completed ~98% of the training volume. 

### Basal Metabolic Measurements

Resting oxygen consumption (VO_2_), carbon dioxide output (VCO_2_), respiratory exchange ratio (RER), food intake, water intake and spontaneous activity (beam breaks, XAMB) were monitored under a consistent temperature (25°C) using an indirect calorimetry system (Columbus Instruments). Mice were acclimatized to the metabolic chamber for 12h prior to commencing data collection. VO_2_, VCO_2_ and RER were measured in individual mice at 20 minute intervals during a 48h period using a 12h/12 h light-dark cycle with lights on at 0700h. 

### Exercise Testing

The final exercise training session (i.e. 48 hours after the 2^nd^ to last bout of exercise in the training groups) was used to test maximal exercise capacity using a metabolic treadmill (Exer4-Oxymax; Columbus Instruments, Columbus, OH, USA). The test consisted of running on a 10-degree uphill gradient at 11 m/min for 5 min prior to an increase in speed by 2 m/min every 2 min. The experimenter was blinded to the mouse genotype during testing. Volitional exhaustion was defined as spending 10 seconds or more on the shock bar without attempting to re-engage the treadmill. Because the test was performed at an uphill angle, total work performed during the exercise test was calculated as the product of cumulative distance run (meters) and body weight (kg).

### Whole muscle homogenization

Approximately 20 mg of *tibialis anterior* muscle was homogenized in potassium phosphate buffer (50 mM K_2_HPO_4_/KH_2_PO_4_, 1 mM EDTA, 0.1 mM DTT, pH 7.4) supplemented with protease inhibitors (Roche) using an electric homogenizer (Pro Scientific). Insoluble proteins were removed by differential centrifugation at 700×*g*. A portion of the supernatant was supplemented with phosphatase inhibitors (Roche) for use in immunoblotting and both aliquots were stored at -80°C until use. Protein concentration was determined in homogenates with the bichinconic acid method as per the manufacturer’s recommendations (Pierce) with a spectrophotometer (Bio-Rad).

### Mitochondrial Isolations

Mitochondrial fractions were prepared using differential centrifugation. Briefly, ~150 mg of freshly dissected skeletal muscle (*quadriceps femoris*) was homogenized on ice in 1:10 (wt/vol) ice-cold isolation buffer A (10 mM sucrose, 10 mM Tris/HCl, 50 mM KCl, and 1 mM EDTA, and 0.2% fatty acid free BSA, pH 7.4, supplemented with protease inhibitor cocktail (Roche)) using an electric homogenizer (Pro Scientific). The homogenates were then centrifuged for 15 min at 700×*g* and the resulting supernatants were centrifuged for 20 min at 12,000×*g* to pellet mitochondria. All centrifugation steps were carried out at 4°C. Mitochondrial pellets were resuspended in potassium phosphate buffer (50 mM K_2_HPO_4_/KH_2_PO_4_, 1 mM EDTA, 0.1 mM DTT, pH 7.4) plus protease inhibitors (Roche) and, following determination of protein concentration, were used for mtDNA isolation, SDS-PAGE or co-immunoprecipitations.

### SDS-PAGE

Equivalent amounts of protein from each sample were run on acrylamide gels with molecular weight standards, transferred to nitrocellulose and developed using appropriate primary and secondary antibodies. Proteins were separated at 120 volts for approximately 2 hours and then transferred at 110 volts for 1 hour to nitrocellulose membranes (GE Healthcare). Membranes were blocked with milk or bovine serum albumin diluted in tris-buffered saline with tween (TBS-T) for 1 hour and then incubated in primary antibody overnight at 4°C. Antibodies and their dilution used with whole muscle or isolated mitochondria immunoblots are as follows: Anti-Tfam was from Santa Cruz (sc-23588, 1:800). The MitoProfile total OXPHOS (ab110413, 1:1000), anti-nitro tyrosine (ab7048, 1:1000), 4HNE (ab48506, 1:1000), anti-Sod2 (ab13534, 1:3000), anti-Ogg1 (ab204, 1:1000) and anti-UCP3 (ab3477, 1:3000) antibodies were from Abcam. Anti-VDAC #4661, 1:3000 was from Cell Signaling. Anti-ANT1 (MSA02, 1:1000) antibody was acquired from MitoSciences. For determination of protein carbonyls, samples were derivatized and detected using a kit from Millipore (Oxyblot). After primary incubation, all blots were washed 3 times in TBS-T and incubated in respective anti-mouse, anti-rabbit or anti-goat secondary (1:10000; GE Healthcare) antibodies at room temperature for 1 hour. Subsequently, membranes were developed with ECL plus (GE Healthcare) and exposed to x-ray film (GE Healthcare). All films were digitized and band density was determined using ImageJ (NIH).

### Enzyme activities

All enzyme activities were assessed on homogenates that contained protease inhibitors only. Cytochrome c oxidase activity was assayed as described [26], while citrate synthase and complex I + III activity were determined as described [27] with minor modifications: spectrophotometer absorbance was monitored using a continual read for 2 minutes with auto correction for rotenone insensitive reactions using a reference cell.

### Non-enzymatic Antioxidant Capacity

Isolated mitochondrial fractions were analyzed using an assay based on the decolorizing of a solution of 2,2’-azinobis-3-ethylbenzothiazoline-6-sulfonic acid radical cations (ABTS^·+^) by antioxidant solutions as described [28]. All results are expressed as µM Trolox equivalent antioxidant capacity per µg protein (µM TEAC/ µg protein).

### RNA isolation and mRNA analysis

RNA was isolated, reverse transcribed to cDNA and gene expression analyzed by qPCR as described [29]. Primers for mouse *β2-microglobulin* and *Tfam genes* are as follows: *β2-microglobulin*, forward: cccgcctcacattgaaat; *β2-microglobulin*, reverse: gaaagaccagtccttgctgaa; *Tfam*, forward: aacaggacatggaaagcagat; *Tfam*, reverse: gaagggaatgggaaaggtaga.

### Mitochondrial 8-OH-*d*G

mtDNA was isolated from resuspended mitochondria using the QIAamp DNA mini kit (Qiagen). DNA concentration was determined using PicoGreen dye and a lambda DNA standard curve (Invitrogen), followed by digestion to single bases using a one-step Benzonase protocol as described [30]. Digested bases were analyzed for 8-hydroxy-2-deoxyguanosine (8-OH-dG) in duplicate using a commercially available kit (Cayman Chemical) and the amount of 8-OH-dG present in each sample was normalized to initial mtDNA content.

### mtDNA Copy Number Analysis

Total DNA was isolated from 20 mg of *tibialis anterior* muscle using the QIAamp DNA mini kit (Qiagen). The abundance of a mitochondrial DNA gene (COXII) relative to a nuclear gene (β–globin) was determined using quantitative real-time PCR according to the 2^-∆Ct^ method. Primer sequences are as follows: COXII, forward: gccgactaaatcaagcaaca, reverse: caatgggcataaagctatgg; β–globin, forward: gaagcgattctagggagcag, reverse: ggagcagcgattctgagtaga.

### Muscle Fiber Permeabilization and Mitochondrial Respiration

A portion of freshly isolated *quadriceps femoris* muscle was placed into ice-cold permeabilization solution (10 mM Ca^2+^/EGTA buffer, 0.1 μM free calcium, 5.77 mM Na_2_ATP, 6.56 mM MgCl_2_, 20 mM Taurine, 15 mM Na_2_Phosphocreatine, 20 mM Imidazole, 0.5 mM DTT, and 50 mM MES; BIOPS) to isolate fiber bundles as described [31]. Muscle was carefully dissected free of any fat and connective tissue and separated into bundles under a dissection microscope with fine forceps for 20 minutes. Fibers were then collected, immersed in fresh BIOPS solution supplemented with saponin (50 μg/ml) and incubated at 4°C on a rotator for 30 mins. Bundles were then washed twice in respiration buffer (0.5 mM EGTA, 3 mM MgCl_2_, 60 mM K-lactobionate, 20 mM taurine, 10 mM KH_2_PO_4_, 20 mM HEPES, 110 mM sucrose, and 1 g/L BSA (fatty acid free), pH 7.1) for 5 mins on a rotator at 4°C to remove any residual permeabilization solution. Permeabilized fiber bundles were blotted dry, weighed (1-2 mg) and transferred to a high-resolution respirometer (Oroboros Instruments) containing air saturated respiration buffer at 37°C and the chamber was closed. Standardized calibrations to correct for background oxygen flux were completed prior to performing the experimental procedures. All experiments were performed in duplicate, simultaneously.

Basal respiration without adenylates was obtained by the addition of 10 mM glutamate and 2 mM malate as substrates of complex I. This was followed by injection of 2.5 mM ADP to assess oxidative phosphorylation through complex I. The subsequent addition of succinate (10 mM) provided the measurement of convergent electron flux through complexes I and II. Mitochondrial outer membrane intactness was tested by the addition of 10 μM cytochrome c; no change in respiration was observed in our preparations in the presence of cytochrome c. Experiments were performed at oxygen concentrations greater than 80 μM to prevent diffusion limitations present in permeabilized fibers. Measurements were acquired using steady state regions of oxygen flux following substrate addition.

### Co-immunoprecipitation

Isolated mitochondria lysates (200 μg) were solubilized using 1% n-dodecyl maltoside prior to performing immunoprecipitations and stored at -80°C. Subsequently, 20 μl of protein A/G plus agarose beads was added to spin columns (Pierce), washed twice with PBS, washed twice with 200 μl of wash buffer (PBS/0.05% n-dodecyl maltoside with protease inhibitors (Roche)), spun, and incubated with 2 μg of anti-TFAM antibody (Santa Cruz, sc-23588) in PBS for 2 hours at 4°C to conjugate the antibody to the beads. After 2 additional washes in wash buffer, the solubilized mitochondrial lysate was added to the columns and allowed to rotate overnight at 4°C. The following morning, the columns were spun to collect the proteins not bound to the beads (IP supernatant). This fraction did not contain any detectable TFAM on western blots, indicating that all of the TFAM protein had been depleted from the original sample (Figure S3A). After collecting the supernatant, the beads were washed twice with 200 μl of wash buffer, plugged, and incubated with 50 μl of 1% SDS and spun to elute any immunoprecipitated proteins. A second elution of SDS produced no detectable TFAM protein from the beads, indicating complete extraction. All spins were done at 3,000 rpm for 1 minute at 4°C. The immunoprecipitates were run on gels using SDS-PAGE and transferred to nitrocellulose membranes. Membranes were probed using anti-TFAM (Cell Signaling #7945), anti-GRP75 (Abcam, ab2799) and anti-HSP60 (Abcam, ab59457) to detect the amount of HSP60 and GRP75 associated with TFAM according to the SDS-PAGE methods. No signal was present when using an IgG control antibody for immunoprecipitations (Figure S3B). Additionally, only TFAM was detected in sample that had been heat denatured at 95°C prior to performing the TFAM immunoprecipitation, indicating that the HSP60 and GRP75 protein detected in the immunoprecipitates was due to a specific protein-protein interaction with TFAM (Figure S3C).

### 2-dimensional Blue Native PAGE

Mitochondrial lysates (50 μg) were sedimented by centrifugation at 19,200×*g* for 10 minutes at 4°C. After removing the supernatant, mitochondria were solubilized in 40 μl of 0.75 M aminocaproic acid/0.05 M Bis-tris followed by the addition of 7.5 μl of 10% n-dodecyl lauryl maltoside based on the described method [32]. Additionally, prior to gel loading, 2.5 μl of 0.05% coomasie blue/1M aminocaproic acid was added to each sample. Samples and a native protein standard ladder (Invitrogen) were loaded onto a single 10% first dimension native acrylamide gel with an empty lane separating samples and run overnight at 100 volts at 4°C. After the gel run, sample lanes were excised from the gel, soaked in denaturing buffer (10% glycerol, 2% SDS, 50 mM Tris (pH 6.8), 50 mM DTT, and 0.002% Bromophenol blue) turned 90 degrees and placed in an open well at the top of a 12.5% second dimension SDS-PAGE gel. The native ladder was soaked in coomasie blue and destained until bands were visualized in order to identify the extent of native mitochondrial complex migration. A pre-stained molecular weight ladder was also run in the second dimension SDS-PAGE gel. Blots were transferred to nitrocellulose membranes at 110 volts for 1 hour and then blocked in 5% milk for 3 hours. Subsequently membranes were incubated in a mitochondrial cocktail primary antibody that recognizes a subunit of each of the 5 complexes of the electron transport chain (MitoProfile total OXPHOS antibody; ab110413, 1:1000 in 1% milk/TBS-T) overnight at 4°C. Complex 2 was not well visualized using this relatively low amount of protein and thus was not included in the results. Blots were then incubated in an anti-mouse HRP conjugated secondary antibody and visualized using ECL.

### Statistical Analyses

Baseline comparisons prior to initiating exercise training were assessed using an unpaired *t*-test, with significance accepted as *p* < 0.05. At the conclusion of the exercise study, groups were compared using a 2-way ANOVA with training status (SED vs. EX) and genotype (*Sod2* ^*+/-*^ vs. *Sod2* ^*+/+*^) as factors. Significance was accepted as *p* < 0.05. When differences were observed, the indicated effects were analyzed using a Tukey’s HSD post-hoc test.

## Results

### Sedentary *Sod2* ^*+/-*^ mice have equivalent baseline running performance to wild-type mice, but fail to augment work capacity following exercise training

**Figure 1:**
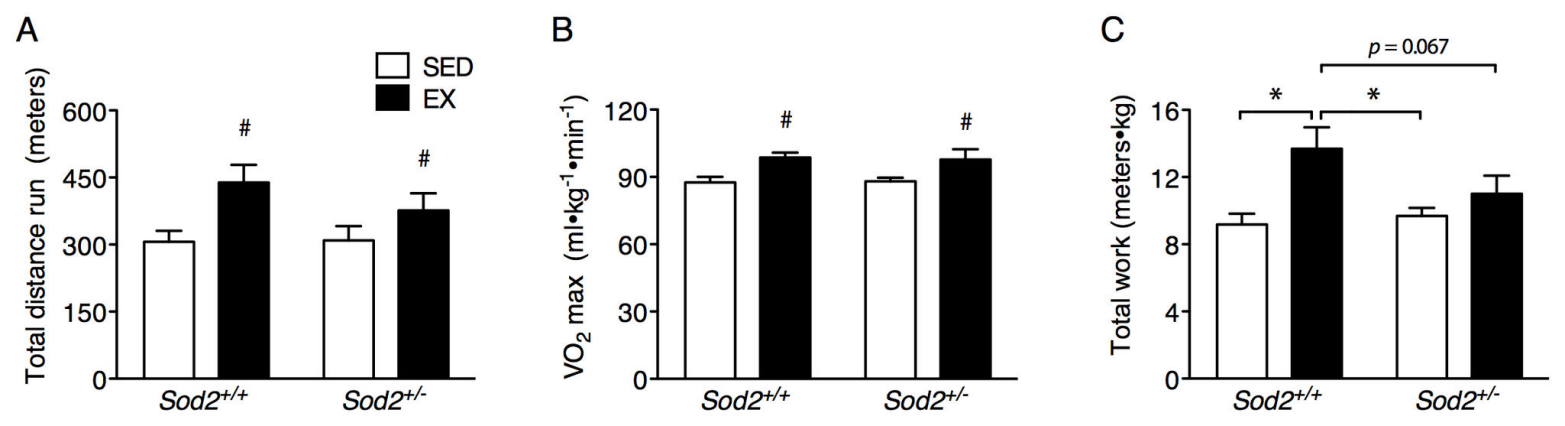
Elevated mitochondrial oxidative stress impairs the improvement in work capacity resulting from exercise training. Mice were tested for exercise capacity on a treadmill at a 10-degree angle after 4 months of exercise training or remaining sedentary as stated in the Materials and Methods. (A) Total distance run, (B) VO_2max_, and (C) total work performed during an exercise test to exhaustion. (For resting data regarding body weight, VO_2_, VCO_2_, food intake and ambulatory activity see Figure S1). Data are mean±SE. *n* = 7-8. *Indicates a significant difference (*p* < 0.05) from the indicated group as determined by ANOVA. ^***#***^Indicates a main effect of exercise training.

In order to study the specific effects of mitochondrial oxidative stress on exercise adaptations, we monitored *Sod2* ^*+/+*^ and *Sod2* ^*+/-*^ mice at before and after initiating exercise training. We found no differences between genotypes with regard to basal metabolic and behavioral parameters (body weight, VO_2_, VCO_2_, food intake and accumulated x-axis ambulatory activity) prior to or following the exercise training (Figure S1A-J). However, after mice had been separated into sedentary (SED) or forced-exercise training (EX) groups for four months, Sod2^+/+^ and *Sod2* ^*+/-*^ EX mice improved several aspects of running performance (total distance run and VO_2max_, Figure 1A-B) compared to their sedentary counterparts, however *Sod2* ^*+/-*^ mice did not exhibit an exercise-induced improvement in work capacity (Figure 1C). A change in maximal oxygen uptake (VO_2_) without an alteration in work capacity in the *Sod2* ^*+/-*^ EX mice suggests adaptive defects in energy generation; therefore we next chose to assess how mitochondrial function was impacted in each genotype with exercise training.

### Mitochondrial function is disrupted in *Sod2* ^*+/-*^ mice and fails to improve in spite of an exercise-induced increase in mitochondrial protein content

**Figure 2:**
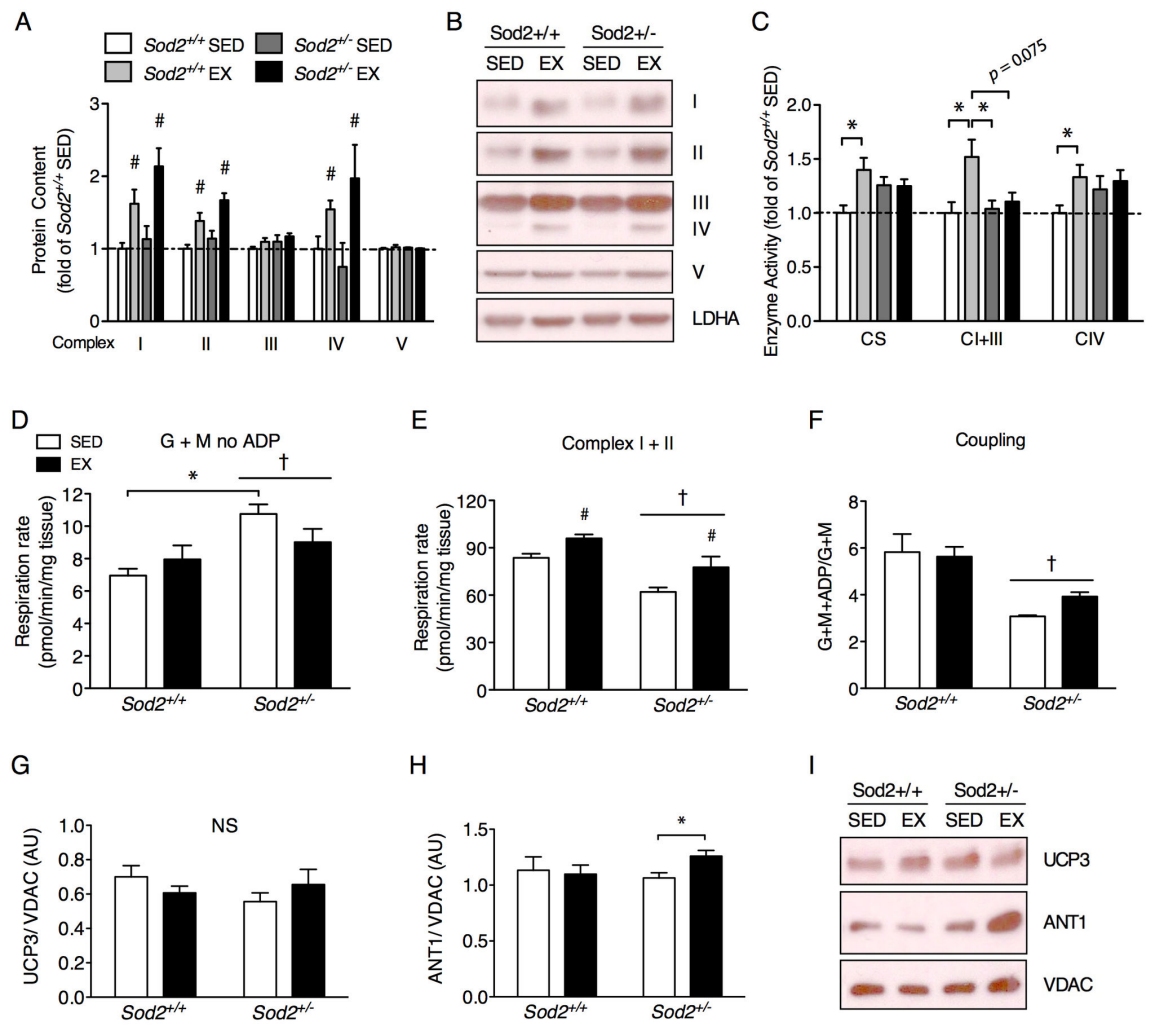
Mitochondrial function is compromised with exercise training in *Sod2* ^*+/-*^ mice without influencing organelle biogenesis. Total protein lysates of tibialis anterior muscle were probed by immunoblotting for individual complex subunits (I: NDUFB8, II: SDHB, III: UQCRC2, IV: COXI, V: ATP5A) or assayed for enzyme activity. (A) The protein expression of mitochondrial proteins determined by densitometry relative to LDHA, (B) representative immunoblots of mitochondrial proteins and (C) enzyme activities of citrate synthase (CS), complex I+III, and complex IV (CIV) per unit of protein (*n* = 8-10). Data are expressed relative to the Sod2^+/+^ SED group. Mitochondrial respiration rates in saponin permeabilized muscle fibers from quadriceps femoris muscle using (D) glutamate and malate without adenylates to indicate a respiratory leak state, (E) glutamate, malate, succinate and ADP to measure maximal coupled oxidative phosphorylation capacity and (F) respiratory coupling ratio of ADP stimulated and non-stimulated respiration using glutamate and malate as substrates (*n* = 4-5). (G) UCP3 and (H) ANT1 protein expression in isolated mitochondrial lysates from quadriceps femoris muscle relative to VDAC and (I) representative immunoblots for each group (*n* = 8-10). All data are mean±SE. *Indicates a significant difference (*p* < 0.05) from the indicated group as determined by 2-way ANOVA. ***#***Indicates a main effect of exercise training (*p* < 0.05). ***^†^***Indicates a main effect of genotype (*p* < 0.05). NS, non-significant.

Oxidative stress has been shown to induce mitochondrial biogenesis *in vitro* to compensate for impaired mitochondrial function [33]; however, we did not find basal differences in skeletal muscle mitochondrial protein content or enzyme activity between *Sod2* ^*+/+*^ and *Sod2* ^*+/-*^ SED mice (Figure 2A-C), consistent with the similar SED group exercise performance (see Figure 1A-C). However, exercise training resulted in a similar increase in the protein content of complex I, II, and IV in both genotypes (Figure 2A-B) while complex III and V remained unchanged. Nevertheless, the maximal activity of the mitochondrial proteins citrate synthase, complex I+III, and complex IV were augmented only in *Sod2* ^*+/+*^ mice with exercise (Figure 2C), suggesting that mitochondrial oxidative stress in *Sod2* ^*+/-*^ mice did not alter the exercise-induced augmentation of mitochondrial protein expression, yet it did significantly disrupt their assembly into functional complexes. Further, mitochondrial respiration in permeabilized muscle fibers from *Sod2* ^*+/-*^ mice was generally elevated compared to *Sod2* ^*+/+*^ mice in the presence of glutamate and malate without ADP addition (Figure 2D), which is indicative of proton leak or redox electron transport “slip”. Maximal coupled respiration (complex I+II substrates with ADP addition) increased with training in both genotypes but remained lower overall in Sod2^+/-^ mice compared to Sod2^+/+^ mice (Figure 2E), which is likely reflected in the blunted whole body work capacity in the *Sod2* ^*+/-*^ EX mice (see Figure 1C). Additionally, there was generally lower respiratory control in *Sod2* ^*+/-*^ mice, but this was not affected by exercise training (Figure 2F). 

Given the normal increase in mitochondrial proteins observed in *Sod2* ^*+/-*^ EX mice, but the failure of this group to similarly normalize mitochondrial function, we sought to characterize mitochondrial uncoupling proteins that dissociate oxygen utilization and ATP generation [34]. However, UCP3 protein content per amount of mitochondria was unaltered in the current study (Figure 2G) and because there were no differences in resting metabolic rate between genotypes after the training intervention (Figure S1F-J), there is little support for a role of UCP3 in impairing the oxidative capacity of the *Sod2* ^*+/-*^ EX mice. However, there were differences in the non-ADP stimulated “leak state” of mitochondrial respiration between *Sod2* ^*+/-*^ and *Sod2* ^*+/+*^ SED mice (see Figure 2D), so it is possible that UCP3 or some other uncoupling mechanism may play a minor role in untrained muscle in a preliminary attempt to deal with excess oxidative stress. 

Adenine nucleotide translocase 1 (ANT1) can also facilitate uncoupling by allowing protons to leak across the mitochondrial inner membrane [35] and we found that ANT1 content was elevated in response to exercise training solely in *Sod2* ^*+/-*^ mice (Figure 2H). ANT1 proton conductance is related to its content, but not its ADP/ATP translocase activity [35]. ANT1 did not differ in its basal expression between SED *Sod2* ^*+/+*^ and *Sod2* ^*+/-*^ mice and since reduced coupling was observed in both *Sod2* ^*+/-*^ SED and EX mice (compared to *Sod2* ^*+/+*^) it seems unlikely that the training-induced elevation in ANT1 accounts for the lack of aerobic adaptation in the *Sod2* ^*+/-*^ mice. It is probable that elevated ANT1 is at least partly compensating for impaired ATP generation in the *Sod2* ^*+/-*^ EX group due to upstream mitochondrial complex dysfunction. Overall, these results indicate that increasing mitochondrial protein expression in concert with elevated oxidative stress seems to exacerbate minor mitochondrial dysfunction per unit of mitochondrial protein rather than correct it.

### Exercise in *Sod2* ^*+/-*^ mice does not abrogate mitochondrial oxidative damage

**Figure 3:**
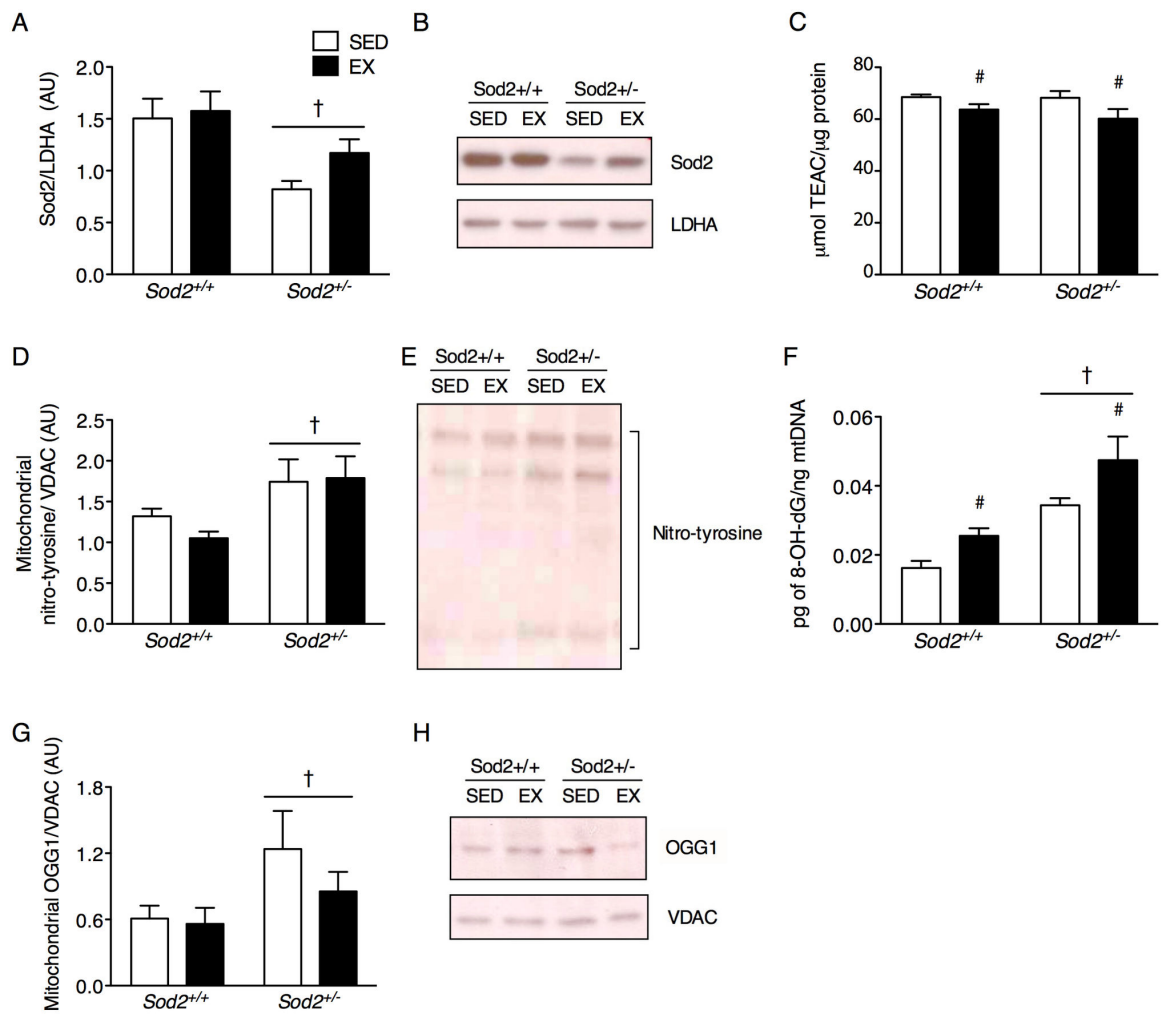
Mitochondrial oxidative stress persists with exercise training in *Sod2* ^*+/-*^ mice. Total protein lysates were prepared from tibialis anterior muscle and isolated mitochondrial lysates from quadriceps femoris muscle (A) Sod2 protein in total lysates relative to LDHA and (B) representative immunoblots. (C) Non-enzymatic anti-oxidant capacity of mitochondrial lysates per amount of protein measured expressed as Trolox Equivalent Antioxidant Capacity (μmol TEAC/μg protein). (D) Quantification of mitochondrial nitro-tyrosine and (E) representative immunoblots. For the results of the non-significant oxidative damage modifications 4HNE and protein carbonyls, see Figure S2A-B. (F) Levels of the oxidative lesion 8-hydroxy-2-deoxyguanosine in isolated mitochondrial DNA digested to single bases. (G) Expression of the DNA repair enzyme OGG1 in isolated mitochondrial lysates relative to VDAC and (H) representative immunoblots. *n* = 8-10 per group for all measurements. All data are mean±SE. ***#***Indicates a main effect of exercise training (*p* < 0.05). ***^†^***Indicates a main effect of genotype (*p* < 0.05).

Higher levels of oxidative damage are found in association with several age-associated disorders and instances of mitochondrial dysfunction [5,6]. Exercise is known to stimulate compensatory mechanisms to alleviate oxidative stress including the expression of antioxidant enzymes, the import of non-enzymatic antioxidant compounds (i.e. vitamin C, E) and signaling repair mechanisms in response to macromolecular and DNA oxidative damage [36,37]. As expected, Sod2 protein in skeletal muscle was generally lower in *Sod2* ^*+/-*^ mice (Figure 3A-B). Since prior work has demonstrated minor defects in cytosolic antioxidant enzymes within the muscle of *Sod2* ^*+/-*^ mice [38], we sought to account for any differences in non-enzymatic radical scavenging ability (i.e. as occurs with vitamin E) by measuring the reactivity of isolated mitochondrial lysates with the ABTS cation in comparison to the standard compound Trolox. We determined that exercise generally reduced non-enzymatic antioxidant activity in isolated mitochondria (Figure 3C), a phenomenon that would impair free radical scavenging and be particularly detrimental within *Sod2* ^*+/-*^ muscle. The typical exercise stimulated antioxidant mechanisms were clearly ineffective in mitigating mitochondrial oxidative stress as nitro-tyrosine adducts were generally elevated in *Sod2* ^*+/-*^ mice regardless of training group (Figure 3D-E). Importantly, while nitro-tyrosine levels per amount of mitochondria were similar between the *Sod2* ^*+/-*^ SED and EX groups, the EX group had ~40% more mitochondria on the whole cell level implying a higher absolute burden of oxidatively modified proteins. Previous work has shown protein carbonyl levels to be elevated in *Sod2* ^*+/-*^ mice in liver mitochondria [39] and in aged skeletal muscle [40], but neither the lipid peroxidation product 4-hydroxy-nonenal or protein carbonyls were elevated in *Sod2* ^*+/-*^ skeletal muscle mitochondria in the current study (Figure S2A-B). However, isolated mitochondria from skeletal muscle exhibits higher complex I, II, III and IV activity than liver [41] and may be susceptible to different cellular radicals because it is predominantly terminally differentiated. 

In parallel to the elevated nitro-tyrosine levels in mitochondria, *Sod2* ^*+/-*^ mice had greater levels of 8-hydroxy-2-deoxy guanosine (8-OH-dG) damage to mtDNA (Figure 3F) compared to *Sod2* ^*+/+*^ mice. Surprisingly, exercise training increased 8-OH-dG in both genotypes. While the modification of mtDNA is a common consequence of oxidative stress, exercise training has previously been shown to reduce total 8-OH-dG levels, often in concert with increased antioxidant enzyme expression [26,42]. However, the absolute measurement of mitochondrial 8-OH-dG levels in skeletal muscle is rare. In agreement with the 8-OH-dG analysis, the mitochondrial expression of the base excision repair enzyme OGG1 was higher in *Sod2* ^*+/-*^ compared to *Sod2* ^*+/+*^ mice (Figure 3G-H).

### Mitochondrial oxidative stress disrupts mtDNA transcription by altering the stoichiometry of mtDNA copies:TFAM protein

**Figure 4:**
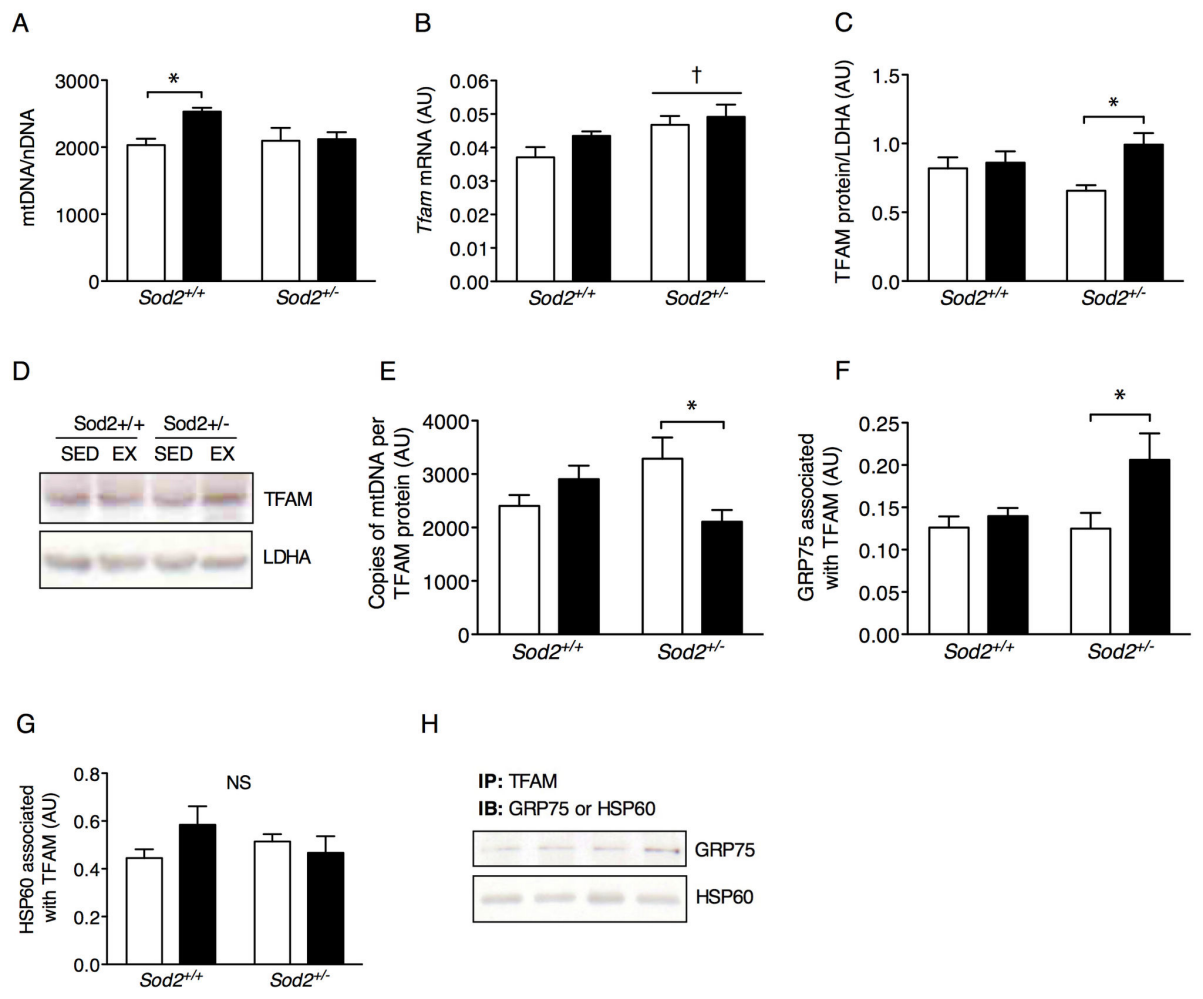
mtDNA transcription is disrupted by oxidative stress due to impaired TFAM function. (A) Mitochondrial DNA copy number in tibialis anterior muscle relative to copies of genomic DNA (mtDNA/nDNA; *n* = 8-10). (B) *Tfam* mRNA expression using β2-microglobulin as the housekeeping gene in quadriceps femoris muscle, (C) TFAM protein content relative to LDHA in tibialis anterior muscle and (D) representative immunoblots (*n* = 8-10). (E) Copies of mtDNA expressed relative to TFAM protein (*n* = 8-10). (F) GRP75 and (G) HSP60 protein co-immunoprecipitated with TFAM in isolated mitochondria and (H) representative immunoblots for each group (*n* = 3, for control experiments see also Figure S3A-C). All data are mean±SE. *Indicates a significant difference (*p* < 0.05) from the indicated group(s) as determined by ANOVA. †Indicates a main effect of genotype (*p* < 0.05).

mtDNA is vulnerable to oxidative damage from byproducts of mitochondrial electron transport and this is exacerbated by its lack of protective histones. Mitochondrial transcription factor A (TFAM) is essential for the transcription and maintenance of mtDNA and is believed to coat a significant portion of its length at all times [43]. Exercise training predictably induced an increase in mtDNA copy number in *Sod2* ^*+/+*^ but this did not occur in *Sod2* ^*+/-*^ mice (Figure 4A). Conversely, *Tfam* mRNA was basally elevated in *Sod2* ^*+/-*^ mice (Figure 4B) and TFAM protein expression in whole muscle was only increased in *Sod2* ^*+/-*^ mice following exercise training (Figure 4C-D). This indicates some degree of compensation in TFAM protein expression, possibly related to altered mtDNA stability. Consequently, we expressed mtDNA relative to TFAM protein and found this ratio was reduced in *Sod2* ^*+/-*^ EX mice vs. *Sod2* ^*+/-*^ SED (Figure 4E). Since recombinant TFAM binds less efficiently to synthesized 8-OH-dG DNA adducts [44], and because 8-OH-dG damage to mtDNA is increased with exercise training in *Sod2* ^*+/-*^ mice, greater expression of TFAM protein might be an attempt to preserve mtDNA integrity. However, TFAM-bound mtDNA may also prevent base excision repair enzymes such as OGG1 from accessing mtDNA [45] possibly explaining the reduced OGG1 expression in *Sod2* ^*+/-*^ EX vs. *Sod2* ^*+/-*^ SED mice (see Figure 3G-H). Moreover, the activity of the mitochondrial Lon protease, which catabolizes TFAM, has been reported to be lower in *Sod2* ^*+/-*^ mice [40] and thus damaged or malfunctioning TFAM may be incompletely removed in the presence of mitochondrial ROS damage. 

In order to assess whether TFAM had been modified by oxidative stress, TFAM was immunoprecipitated from isolated mitochondrial lysates. However, there were no detectable nitro-tyrosine adducts present on TFAM which might explain its altered function (data not shown). Alternatively, TFAM misfolding could affect its DNA binding activity, and thus we probed for mitochondrial chaperone proteins that were potentially bound to TFAM in response to oxidative stress. We found more of the chaperone GRP75, but not HSP60, associated with TFAM in the *Sod2* ^*+/-*^ EX vs. SED mice (Figure 4F-H), possibly reflecting a response to sequester dysfunctional TFAM molecules that evaded proteolysis. This appears to be a targeted chaperone response rather than a general unfolded protein response as GRP75 was not altered in isolated mitochondria (data not shown). mtDNA transcription therefore appears sensitive to local oxidative stress, which may explain reduced mtDNA copy number in pathologies associated with mitochondrial ROS damage such as COPD or Alzheimer’s disease [46,47].

### Electron transport chain complexes from oxidatively damaged mitochondria exhibit increased protein misfolding and mis-assembly

**Figure 5:**
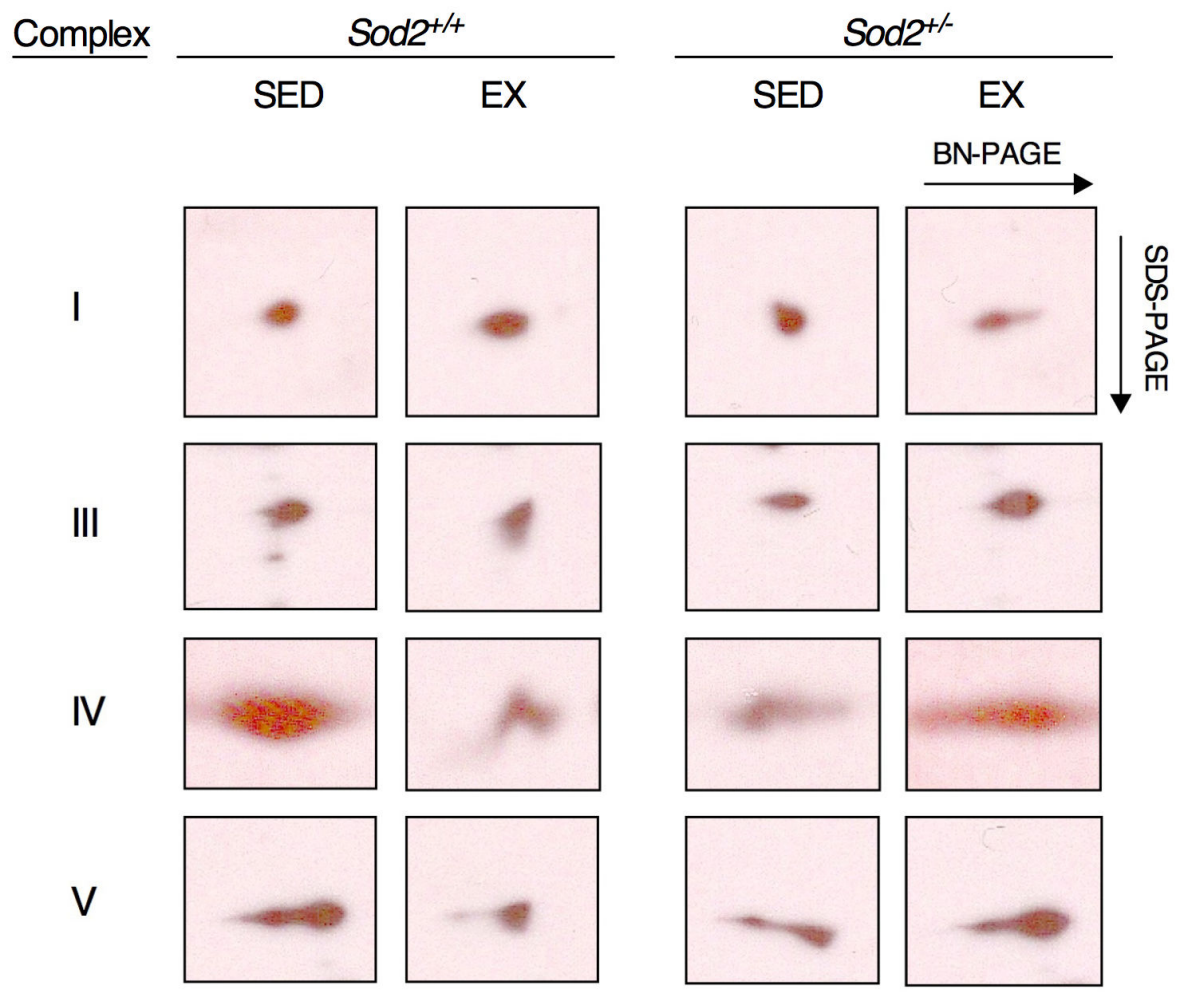
Mitochondrial complex assembly worsens in *Sod2* ^*+/-*^ mice subjected to exercise. Lauryl-maltoside solubilized mitochondria were separated using two-dimensional Blue-Native PAGE in order to visualize native migration of each of four electron transport chain complexes. A lack of intermediate species in assembling the native complex is visualized as a shorter horizontal migration, while unassembled complexes are evident as tails or very broad bands. Note the divergent response to exercise between the two genotypes. Samples were run simultaneously on the same native gel. Image frames are identically sized for each complex and were acquired from the same film exposure. This experiment was repeated twice using different samples from each of the four groups in order to confirm that this was a reproducible observation.

Since mitochondrial protein function is clearly disrupted in the *Sod2* ^*+/-*^ SED mice and exacerbated in the *Sod2* ^*+/-*^ EX mice, we sought to examine whether ETC complex assembly contributed to the impairments in exercise adaptation. Exercise training generally appeared to improve the native assembly of the ETC complexes I, III, IV and V in *Sod2* ^*+/+*^ mice as immunoblotting indicated more punctate migration in the native direction in EX vs. SED mice (Figure 5). Conversely, exercise training appears to worsen complex formation in the *Sod2* ^*+/-*^ mice, particularly in the case of complexes I and IV. It is likely that the increased levels of nitrosylated mitochondrial proteins in *Sod2* ^*+/-*^ mice (see Figure 3) interfered with the assembly process within the mitochondrial membrane space. The mis-assembly of mitochondrial complexes is similarly observed in pathologies linked with mitochondrial dysfunction and oxidative stress, including Parkinson’s disease [48] and Alzheimer’s disease [49]. While the causative events of these diseases remain unclear, we propose that excess superoxide can disrupt mitochondrial complex assembly under conditions of mitochondrial expansion or remodeling. 

## Discussion

Mitochondrial oxidative stress has been proposed to contribute to a host of diseases by nature of inducing ETC dysfunction and impairing cellular bioenergetics. We have used *Sod2* ^*+/-*^ mice, which exhibit no overt phenotypic abnormalities in spite of their elevated tissue oxidative damage, to determine if this state of mitochondrial stress alters their ability to respond to a metabolic challenge (exercise). Within this context, we provide evidence of two mechanisms by which excess mitochondrial superoxide impairs mitochondrial function *in vivo* using exercise training as a model to stimulate mitochondrial biogenesis in skeletal muscle: (1) by impeding proper ETC complex assembly and (2) via disruption of mtDNA copy number.

While excess mitochondrial ROS are commonly implicated in the pathogenesis of disease, mice overexpressing *Sod2* do not exhibit signs of a metabolic advantage within differentiated skeletal muscle or in lifespan [50,51]. These data, in combination with reports suggesting that antioxidants disrupt mitochondrial exercise adaptation [12,13], imply that mitochondrial antioxidants may only be relevant to mitochondrial adaptation in deficient states. However, it remains possible that supplemented “antioxidants” used in these studies actually acted as pro-oxidants *in vivo* and compromised metabolic function in skeletal muscle via the mechanisms observed in the current study or via alternative oxidative modifications. Furthermore, the current findings demonstrating higher oxidative damage following exercise training (see Figure 3F) and the superoxide mediated disruption of mtDNA transcription (see Figure 4A) suggest caution in the implementation of exercise interventions in populations that might have persistent mitochondrial oxidative stress or that might be sensitive to mitochondrial stressors. While in the current study we did not measure any pathology or longevity outcomes, these types of analyses will be necessary in the future in order to more accurately determine the physiologic consequences of compromised antioxidant defenses in the context of exercise or other types of metabolic challenge. 

## Supporting Information

Figure S1 **Metabolic and behavioral measurements before and after separation into study groups.** Initial measurements of (A) body weight, (B) VO_2_, (C) VCO_2_, (D) total food intake and (E) X-axis ambulatory activity during light and dark cycles in Sod2^+/+^ and Sod2^+/-^ mice. Measurements in the same mice after separation into groups of (F) body weight (G) VO_2_, (H) VCO_2_, (I) total food intake and (J) X-axis ambulatory activity. Data are mean±SE and contain no significant differences between groups.
(PDF)

Figure S2 **Non-significant mitochondrial oxidative damage analysis.** Densitometry and immunoblots of (A) 4-Hydroxynonenal (4HNE) normalized to VDAC and (B) protein carbonyls in isolated mitochondria from *quadriceps* *femoris* muscle in *Sod2* ^*+/+*^ and *Sod2* ^*+/-*^ SED and EX mice. Data are mean±SE. NS, non-significant.
(PDF)

Figure S3 **Immunoprecipitation (IP) control experiments.** (A) The extent of TFAM depletion from mitochondrial lysate by comparing the sample after IP (IP-SN) with the pulled-down sample in terms of TFAM content. (B) Immunoprecipitation with anti-TFAM antibody or IgG control antibody to confirm that no non-specific interactions occurred. (C) Normal Immunoprecipitation of TFAM using normal conditions (IP) and with heat-denaturing of the sample at 95°C for 5 minutes prior to immunoprecipitation (IP+HD) to confirm that the signal on immunoblots was due to a specific protein-protein interaction with TFAM.
(PDF)
