# Supplementary material for: Elevated Mitochondrial Oxidative Stress Impairs Metabolic Adaptations to Exercise in Skeletal Muscle
Source: PLoS One. 2013 Dec 6;8(12):e81879. doi: 10.1371/journal.pone.0081879 (PMC3855701; doi:10.1371/journal.pone.0081879)

Fig. S1

Basal measurements, before training intervention

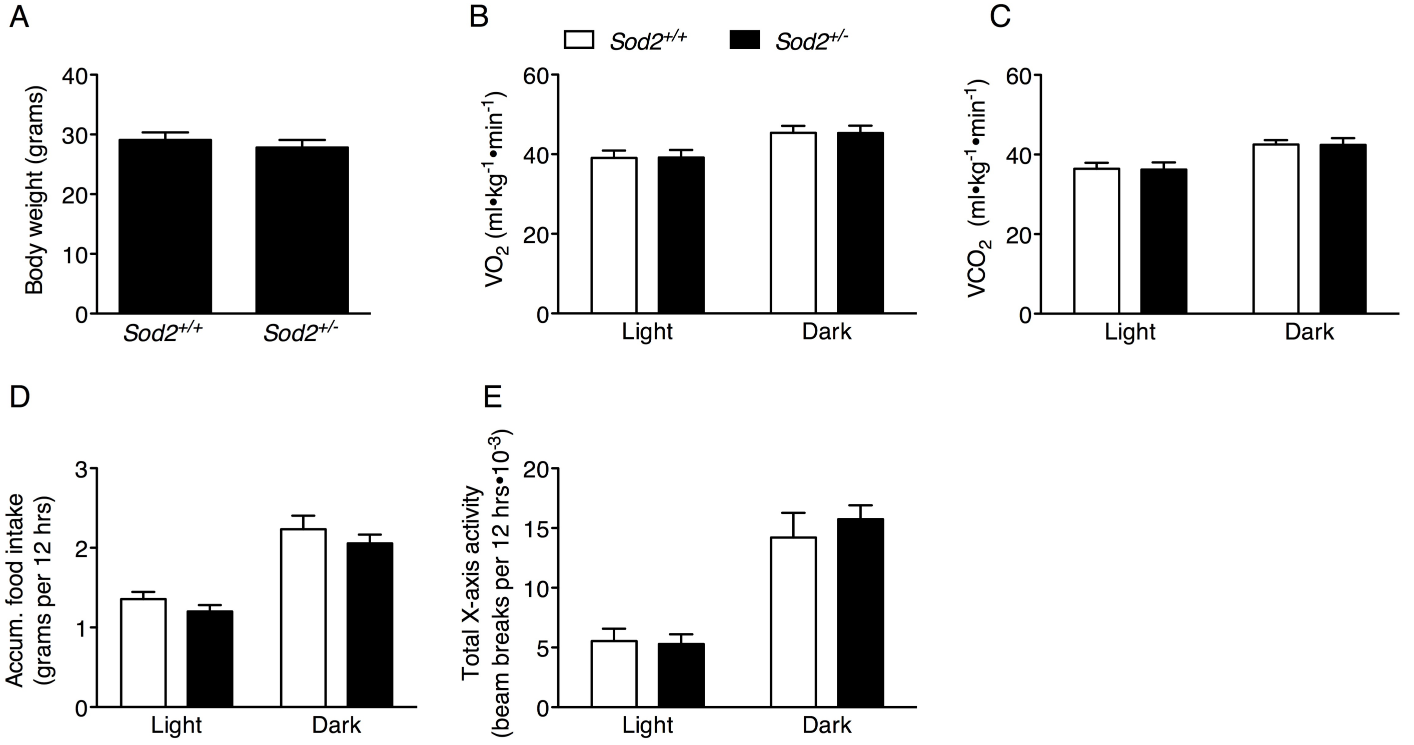

Basal measurements, after training intervention

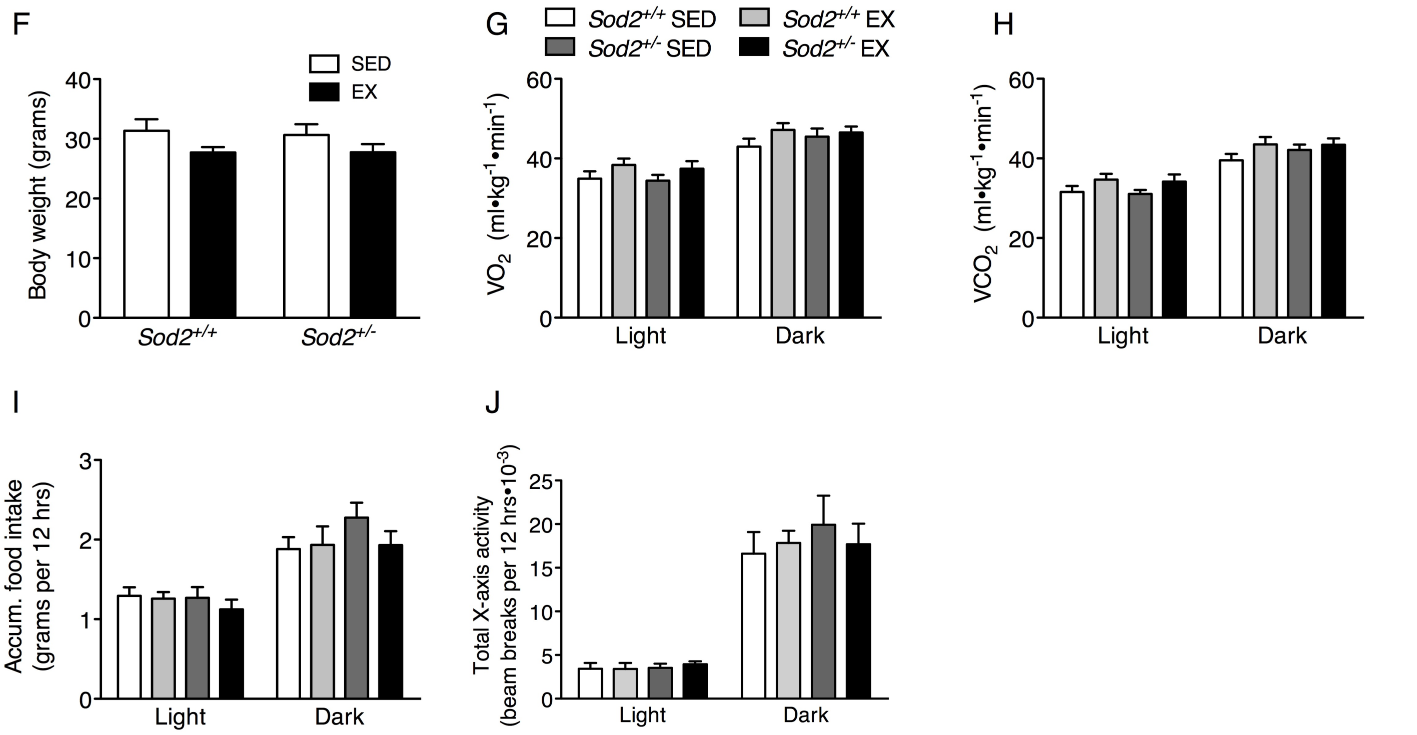

Supplement: Figure S1 — Metabolic and behavioral measurements before and after separation into study groups. Initial measurements of (A) body weight, (B) VO2, (C) VCO2, (D) total food intake and (E) X-axis ambulatory activity during light and dark cycles in Sod2+/+ and Sod2+/- mice. Measurements in the same mice after separation into groups of (F) body weight (G) VO2, (H) VCO2, (I) total food intake and (J) X-axis ambulatory activity. Data are mean±SE and contain no significant differences between groups. (PDF) [file pone.0081879.s001.pdf]
