# Supplementary figures and images for: Elevated Mitochondrial Oxidative Stress Impairs Metabolic Adaptations to Exercise in Skeletal Muscle
Source: PLoS One. 2013 Dec 6;8(12):e81879. doi: 10.1371/journal.pone.0081879 (PMC3855701; doi:10.1371/journal.pone.0081879)

Fig. S2

A

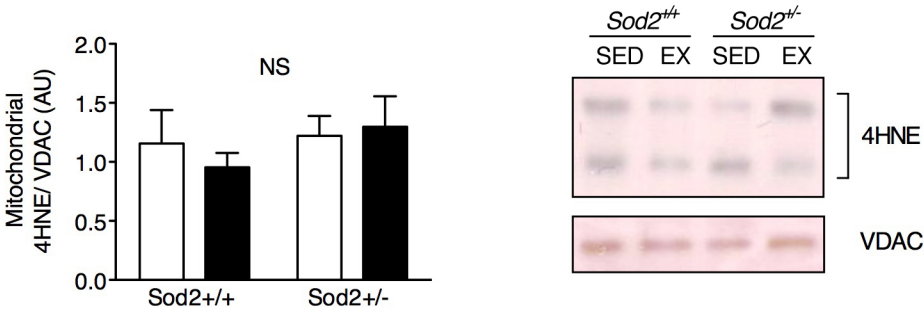

B

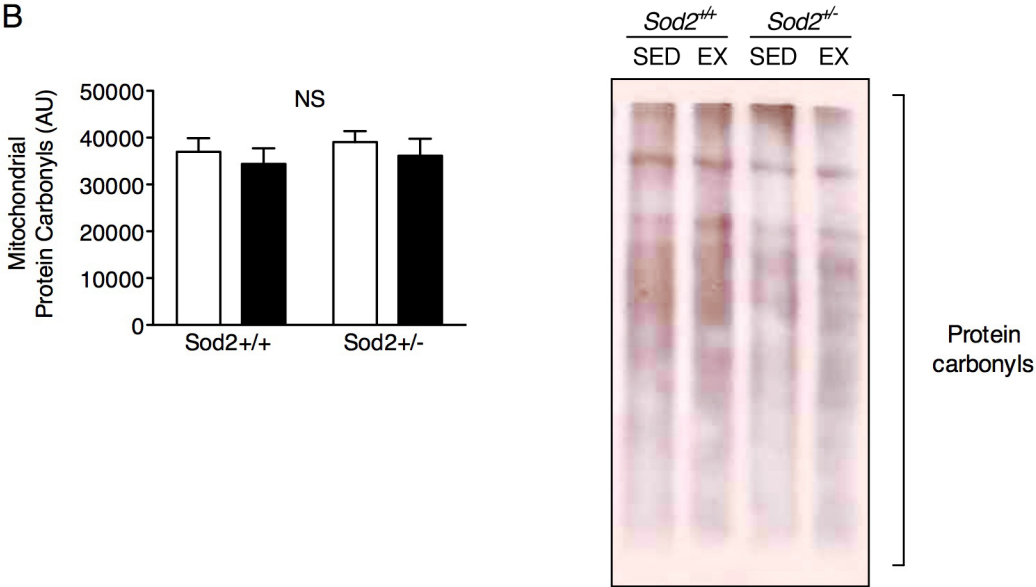

Supplement: Figure S2 — Non-significant mitochondrial oxidative damage analysis. Densitometry and immunoblots of (A) 4-Hydroxynonenal (4HNE) normalized to VDAC and (B) protein carbonyls in isolated mitochondria from quadriceps femoris muscle in Sod2 +/+ and Sod2 +/- SED and EX mice. Data are mean±SE. NS, non-significant. (PDF) [file pone.0081879.s002.pdf]

Fig. S3

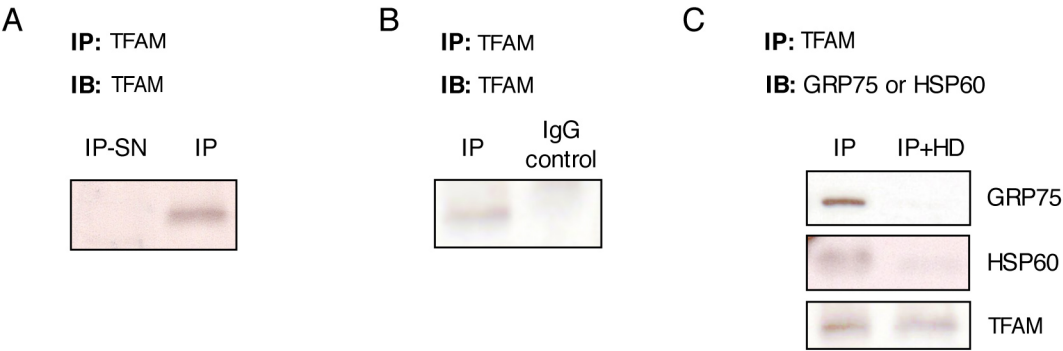

Supplement: Figure S3 — Immunoprecipitation (IP) control experiments. (A) The extent of TFAM depletion from mitochondrial lysate by comparing the sample after IP (IP-SN) with the pulled-down sample in terms of TFAM content. (B) Immunoprecipitation with anti-TFAM antibody or IgG control antibody to confirm that no non-specific interactions occurred. (C) Normal Immunoprecipitation of TFAM using normal conditions (IP) and with heat-denaturing of the sample at 95°C for 5 minutes prior to immunoprecipitation (IP+HD) to confirm that the signal on immunoblots was due to a specific protein-protein interaction with TFAM. (PDF) [file pone.0081879.s003.pdf]
